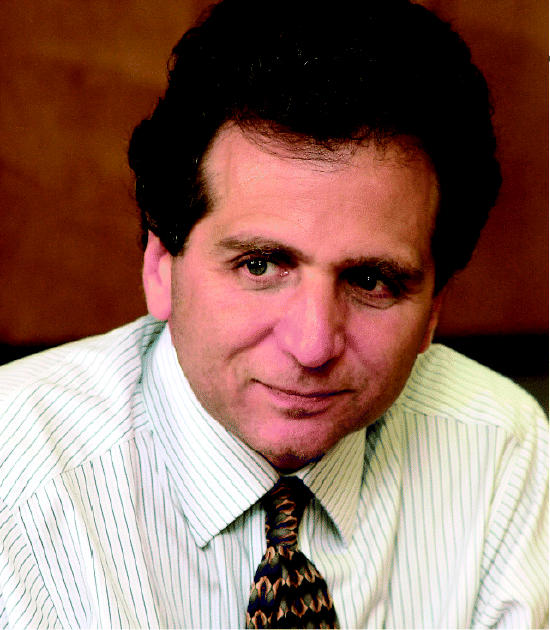# Scientific Vision: Setting Forth a Strategy

**DOI:** 10.1289/ehp.113-a292

**Published:** 2005-05

**Authors:** David A. Schwartz

**Affiliations:** Director, NIEHS, E-mail: schwartzd@niehs.nih.gov

Environmental exposures may adversely affect those who are vulnerable temporally (age, developmental stage), spatially (geographic location), or by unique circumstance (comorbid disease, nutritional status, socioeconomic status, genetics). Understanding the complex relationship between endogenous and exogenous risks within populations and affected individuals, how environmental exposures affect human biology, and how this knowledge can be used to reduce morbidity and extend longevity is precisely the opportunity and challenge that faces the NIEHS. My vision for the NIEHS is to improve human health by increasing this understanding through support of research and professional development in the environmental sciences (toxicology, relevant basic science), environmental medicine, and environmental public health. In addition to understanding how environmental exposures affect human biology, the NIEHS needs to understand how this knowledge can be used to reduce morbidity and extend longevity.

Understanding the complex relationship between endogenous and exogenous risks within populations and affected individuals, how environmental exposures affect human biology, and how this knowledge can be used to reduce morbidity and extend longevity is precisely the opportunity and challenge that faces the NIEHS.

Because environmental exposures contribute substantially to the etiology of many common and complex human diseases, the NIEHS is in a unique position to focus on the interface between environmental exposures, vulnerable populations, human biology and genetics, and the common diseases that limit our longevity. In the postgenomic era of biomedical research, the NIEHS can take a leadership role in improving human health by investigating environmental toxicants to understand how genes work in biological systems, how genetic variants contribute to the development of disease, and why individuals with the same disease have very different clinical outcomes. Moreover, because of its focus and concentrated expertise, the NIEHS is uniquely poised to:

- develop sensitive preclinical markers of exposure and biological response,
- develop strategies to prevent disease in exposed and biologically responsive individuals and populations,
- establish population-based cohorts to understand the impact of environmental exposures on human health,
- understand how environmental exposures affect the course and prognosis of a medical condition, and
- stimulate dialogue to advance our understanding of environmental health policy and ethical issues of environmental concern.

To achieve this vision, I will prioritize individual and programmatic efforts to understand the role of environmental exposures on human health and disease. This will be achieved through the following broad strategies:

- development of interdisciplinary research opportunities that will focus on common, complex diseases with a substantial environmental component;
- efforts to define the epidemiological and clinical significance of environmental exposures in high-risk populations, including those in the international community;
- efforts to understand how genes and genetic variants interact with environmental stimuli to either preserve health or cause disease;
- programmatic integration of basic findings in the environmental health sciences with populations of diseased patients, communities at the extremes of exposure and vulnerability, other academic medical centers, and industry;
- study of environmental toxicants to understand basic mechanisms in human biology;
- use of eukaryotic model systems (yeast, worms, zebrafish, fruitflies, rodents) to accelerate understanding of how environmental exposures affect human health;
- support of the development of high-throughput *in vitro* and *in vivo* bioassays to establish reliable toxicity screens for potential toxicants;
- efforts to strengthen and expand the next generation of environmental health scientists by creating research incentives to encourage basic scientists, epidemiologists, and physician–scientists to develop research careers in the environmental health sciences;
- fostering of an integrated scientific approach that supports partnerships between the NIEHS and other NIH institutes, national and international research agencies, academia, industry, and community organizations to improve human health; and
- support of programs for environmental scientists to work with ethicists and policy makers to fully consider the regulatory implications of our scientific advances in environmental health.

The NIEHS is a complex institute with a distinguished history, a clear purpose, and a dedicated constituency. To develop a plan for fulfilling our mission of improving human health, I will fully engage our dedicated scientific community in a process of strategic planning that will take place over the next year. I will work to involve a broad array of environmental health scientists engaged in toxicology, medicine, epidemiology, public health, basic biology, and genetics in this strategic planning process.

Our field is like no other—we are not limited by a biological system, a disease process, or an organ system. In fact, our scientific discipline represents the critical link between exposure and disease for many other fields of biomedical research. I fully believe that, working together, we can shape the future of the environmental health sciences and realize our critical role in understanding human disease, reducing morbidity, and extending longevity. Our success will be measured in the disease and suffering that we are able to prevent. I feel incredibly privileged to be working with you to meet this challenge, and look forward to your thoughts and comments.

## Figures and Tables

**Figure:**